# The longevity dividend: Why governments and markets must catch up

**DOI:** 10.1113/EP092866

**Published:** 2026-09-02

**Authors:** Nikodem Grzesiak, Serena Kern‐Libera, Richard Faragher, Annalisa Jenkins, Guy Coughlan, Damian Miles Bailey, Carina Kern

**Affiliations:** ^1^ LinkGevity Babraham Research Campus Cambridge UK; ^2^ Centre for Stress and Age‐Related Disease, School of Applied Sciences University of Brighton Brighton UK; ^3^ Genomics England London UK; ^4^ Pensions Institute Bayes Business School London UK; ^5^ Bexorg Inc. New Haven Connecticut USA; ^6^ Neurovascular Research Laboratory, Faculty of Life Sciences and Education University of South Wales Pontypridd UK; ^7^ Life Sciences Working Group European Space Agency Paris France

## 1 THE COMING LONGEVITY DISCONTINUITY

‘How much’ often matters less than ‘how fast’. Financial systems might comfortably accommodate a decade increase in life expectancy if it emerges gradually over half a century, but could they accommodate the same increase within 5 years?

Economies are built to absorb gradual change and are much less comfortable with discontinuities. Human longevity may be approaching that kind of discontinuity. The latest scientific advances point to the emergence of what we have termed ‘system‐level’ therapeutics (Kern, Jenkins et al., 2025; Kern et al., 2024), capable of bringing about rapid, non‐linear changes in both healthspan and lifespan trajectories, challenging the incremental paradigms that underpin current economic and demographic forecasting models. If they do, governments, healthcare systems, insurers and pension funds will have to respond to a break with history, not a continuation of it.

In this paper, we introduce a new conceptual framework that classifies therapeutic interventions according to their biological scope: symptom‐management, single‐disease causal therapeutics and system‐level therapeutics. The first two categories represent the traditional paradigm of medicine, in which therapies are directed at managing symptoms or treating individual diseases. In contrast, the third captures an emerging generation of system‐level interventions that target shared biological mechanisms across multiple age‐related diseases simultaneously (Kern, Jenkins et al., 2025). We outline the expected effects of each category on healthspan, lifespan and downstream economic outcomes. The framework provides a structured way to compare both conventional and the latest emerging therapeutic strategies and to trace how their differing biological impacts propagate through healthcare systems, labour markets, public finances, insurance, and economic productivity.

## 2 FROM DISEASE‐SPECIFIC DRUGS TO SYSTEM‐LEVEL THERAPEUTICS

Blockbuster glucagon‐like peptide‐1 (GLP‐1) receptor agonists, such as semaglutide (Ozempic, Rybelsus and Wegovy) and dulaglutide (Trulicity), together with the dual glucose‐dependent insulinotropic polypeptide (GIP)–GLP‐1 receptor agonist tirzepatide (Mounjaro and Zepbound), are proving to be far more than weight‐loss treatments. Originally introduced for the treatment of type 2 diabetes, these therapies have since expanded to obesity and are now being evaluated for cardiovascular disease and multiple other conditions, with emerging interest in their potential effects on healthy ageing (Ryan et al., 2024; Zheng et al., 2024). Importantly, these benefits are not solely indirect consequences of weight reduction; emerging evidence points to previously unanticipated direct biological effects and novel therapeutic targets (e.g., GLP‐1 may have direct effect on endothelial senescence). This trajectory foreshadows a broader shift in therapeutic potential: from highly indication‐specific drugs focused on symptom management toward mechanism‐based therapies that target conserved biological pathways underlying multiple diseases, addressing fundamental drivers of pathology while delivering benefits that extend across interconnected physiological systems (Kern, Jenkins et al., 2025). Unfortunately, due to an opacity of mechanistic understanding, side effects temper the overall benefits for GLP‐1s in particular, and as recent research demonstrates, there is potential for complications in the form of accelerated ageing phenotypes, from macular degeneration to sarcopenia (Ren et al., 2025; Shor et al., 2025).

While their long‐term impact may take decades to fully emerge, much like antihypertensives, the rapid clinical success of GLP‐1 receptor agonists signals the start of a paradigm shift (Kern, Jenkins et al., 2025). As new data emerge, one thing is clear: we are moving from siloed, disease‐specific targeted treatments toward systemic, potential disease‐modifying therapies. This shift matters. While medicine has long understood that biology operates as an interconnected system, drug discovery has historically pursued a narrow, single‐disease centric focus, contributing to failure rates greater than 90% for clinical drug development on average, and even 100% failure rate for many diseases (Sun et al., 2022).

Kidney disease provides a compelling case study for this transition. Despite the enormous clinical and economic burden of kidney disease, therapeutic innovation has remained remarkably limited, illustrating both the shortcomings of disease‐specific drug discovery and the urgent need for systemic, disease‐modifying approaches. This leaves kidney disease as the ninth leading cause of death worldwide (WHO, 2024). Kidney disease accounts for approximately 24% of annual Medicare expenditure in the United States (around $124 billion), comparable to healthcare spending across Europe (€140 billion). These costs are projected to rise unsustainably over the next decade, with a doubling of NHS expenditure in the United Kingdom alone. This has led to kidney disease being increasingly recognised as a public health emergency (Kidney Research UK, 2023; National Kidney Foundation, 2024). The condition is primarily managed through dialysis or requires a kidney transplant; average life expectancy on dialysis is 5–10 years (Kidney Research UK, 2023; NHS, 2019), and there is a 2.5–3 years waiting time in the United Kingdom for a kidney transplant (Kidney Research UK, 2023) with those who receive a kidney facing an approximate 40% rejection rate within the first 10 years (NHS, 2023).

Faced with staggering failure rates in drug development and the growing burden of age‐related disease such as kidney disease, the traditional model of siloed, single‐disease treatment is no longer sustainable. Systems‐level drugs are far more likely to be effective, given that the chronic diseases which drive ageing and age‐related decline are complex, multifactorial and interlinked. When age‐related diseases share mechanisms and accumulate together, treating them in isolation becomes biologically and economically inefficient. The point is not that kidney disease is uniquely neglected. It is that diseases of ageing share causes, arrive in clusters and interact inside the same person. A therapeutic strategy that ignores those links is fighting the biology one fragment at a time.

But such broad‐spectrum drugs require advances in mechanistic insights. Historically, the Hallmarks of Ageing framework (López‐Otín et al., 2023) has been instrumental in guiding ageing research, identifying ‘features’ such as genomic instability, cellular senescence, and telomere attrition. However, it remains unclear whether these features are primary drivers of ageing, downstream consequences, or simply markers of the ageing process. Now, new integrative tools, in particular such as the Blueprint Theory (Kern et al., 2024) framework, are filling in the gaps of how to map and modulate the fundamental biological mechanisms of ageing itself. By targeting these complex and interconnected pathways, we have the potential to move beyond treating individual diseases to tackling the root causes of systemic decline. Coupled with rapid advances in sophisticated artificial intelligence‐driven analytics, this shift has the potential to upend not only healthcare but also financial and actuarial models (mathematical frameworks used to assess and predict future events in insurance and risk management) that underpin the global economy.

## 3 A PRODUCTIVITY LEVER HIDING IN PLAIN SIGHT

Public debate often treats longevity as a cost curve. That is only half the ledger. In an era of demographic ageing and stagnant economic growth forecasts, one overlooked truth is emerging: longevity science may be the most powerful economic productivity lever of all time.

Governments spend billions annually on education, digital transformation and industrial innovation – all in the name of boosting national productivity. Yet few acknowledge that the single largest drag on workforce participation is declining health in midlife and beyond due to ageing. For example, in the United Kingdom, there are currently more than 2.6 million working‐age people that are out of the labour market due to long‐term illness (The Health Foundation, 2024), resulting in an economic loss of approximately £150 billion annually (Oxera, 2023). Likewise in the United States, the direct costs of chronic health conditions total around $1.1 trillion – equivalent to 5.8% of US gross domestic product (GDP). When indirect costs of lost economic productivity are included, the total costs of chronic diseases in the United States increase to $3.7 trillion – in other words, they equate to almost 20% of the US GDP (Waters & Graf, 2018).

Yet government spending continues to focus on a siloed, disease‐specific approach. For instance, the United Kingdom spend for research seeking to improve ageing is a mere 0.049% of the estimated annual cost of ageing; by comparison, it was suggested that 10% of spending on heart disease goes into research (The Royal Society, 2024). By directly investing in the biology of ageing itself, we have the opportunity to dramatically extend healthy working years, reduce sick leave, prevent premature exits from the labour force, and thus in consequence improve economic return.

Older adults today are not just passive recipients of healthcare; they are caregivers, knowledge bearers, mentors and skilled workers. Every year of productive, healthy life gained adds not only to individual well‐being but also to national economic output. A healthier population over 50 years of age (a decade in which the inactivity rate roughly doubles in the United Kingdom) would relieve pressure on healthcare systems, reduce dependency ratios, and contribute to fiscal stability through extended tax contributions and delayed pension withdrawals. One widely accepted model uses a value of £5 trillion for every extra year of population health in the United Kingdom (Scott et al., 2021).

The economic argument for longevity research is clear: targeting the root causes of ageing is not just a healthcare imperative, it is set to become a non‐negotiable economic strategy. Governments should treat it as such, allocating funding accordingly and incentivising translational research, clinical trials and public–private partnerships in this field. In the 20th century, public investment in antibiotics and vaccines changed the world. In the 21st century, ageing biology has the potential to do the same.

## 4 A CONCEPTUAL FOUNDATION FOR INTEGRATING MECHANISTIC BIOLOGY OF AGEING WITH LONG‐TERM ECONOMIC VALUATION

Current economic models of ageing implicitly assume two conditions: (i) that gains in healthspan and lifespan will occur incrementally, and (ii) that such gains will emerge gradually over time. In other words, conventional projections largely exclude the possibility of a rapid ‘leap in healthy lifespan extension’. These assumptions are historically understandable because economic forecasting has generally extrapolated from prior demographic and medical trends. However, such assumptions may no longer hold, with the emerging effects of GLP‐1 receptor agonists serving as an early harbinger of the shift in drug discovery. There is a realistic possibility that future interventions could produce rapid, non‐linear changes in both healthspan and lifespan trajectories, challenging the incremental paradigms that underpin current economic and demographic forecasting models.

Here, we propose a novel conceptual and quantitative organising structure, designed to integrate mechanistic ageing biology with long‐term economic valuation. The framework provides a formal basis for linking the biological nature of longevity interventions to macroeconomic and societal projections. Critically, it distinguishes therapeutic strategies not simply by disease indication, but by the breadth of their systemic effects on ageing biology itself.

Broadly, therapeutic approaches can be categorised into three groups:

1. **Symptom‐management interventions**. These constitute the majority of medical interventions, where treatment focuses on managing the downstream consequences of pathology rather than modifying underlying ageing processes. Examples include dialysis or transplantation for kidney failure. Such interventions may prolong survival without fundamentally restoring systemic resilience.
2. **Single‐disease causal interventions**. These interventions target a specific disease mechanism and may produce curative or near‐curative outcomes, but remain confined to one pathology. Examples include cisplatin‐based chemotherapy for testicular cancer, which dramatically improved survival when introduced into clinical practice (Chovanec & Cheng, 2022). Although transformative for specific diseases, these interventions generally have limited effects on overall healthspan because ageing individuals accumulate multiple independent pathologies over time.
3. **‘System‐level’ interventions**. This class represents a fundamental paradigm shift in medicine. Unlike the traditional ‘single drug for a single disease’ approach, these therapies target upstream biological drivers shared across numerous age‐related conditions simultaneously. Such interventions aim to modify the ageing system itself, thereby influencing multiple diseases in parallel. In principle, this class of therapeutics has the capacity to produce rapid and substantial gains in both healthspan and lifespan.

At present, clinical medicine is dominated by symptom‐management therapies, while disease‐specific interventions account for most of the remaining therapeutic advances. We are, however, now on the cusp of truly system‐level interventions.

The potential trajectories of longevity‐enhancing therapies are outlined in Table 1 and correspond to these broad categories of therapeutic approaches. The economic implications of these scenarios will vary dramatically depending on whether interventions merely manage symptoms, delay specific diseases or modify fundamental ageing processes.

**TABLE 1:** Potential scenarios.

Scenario	Detail
**Gradual healthspan and lifespan improvement**	This is the trajectory assumed by most traditional actuarial models, with steady, incremental gains in both healthy life expectancy and overall longevity
**Leap in healthy lifespan extension**	In this scenario, people maintain excellent health for much longer periods, forcing the period of ill health further into the future when still more advanced treatments may become available. Advancements in system‐level therapeutics could make this a reality in the near future
**Disproportionate increases in healthspan vs. lifespan**	Longevity interventions need not increase healthspan and lifespan to the same extent. Two contrasting outcomes illustrate the range of possibilities: **Compression of** **morbidity**: Individuals remain healthy for a greater proportion of their lives, with illness compressed into a very short period preceding death. Death still occurs at approximately the same chronological age. In this case, healthspan increases substantially while lifespan changes relatively little **Extension** **of lifespan disproportionate to healthspan**: Lifespan increases while healthspan does not. Biologically, this could occur if life‐limiting diseases are successfully delayed or eliminated while non‐life‐limiting pathologies, including frailty, functional decline and chronic disability, continue to accumulate. Historically considered improbable and is often referred to as the ‘Tithonus error’, after the Greek myth in which Tithonus was granted eternal life without eternal youth and thus condemned to endless ageing. However, this scenario can no longer be dismissed entirely. Advances in medicine, particularly those focused on symptom management and reducing external mortality risks (e.g., accidents and infections), have made it increasingly feasible for individuals to survive despite accumulating non‐lethal pathology. In many respects, modern medicine already operates within this paradigm, managing the consequences of age‐related decline rather than directly addressing its underlying causes. By the age of 50, approximately half of adults in developing countries are expected to experience at least one age‐related chronic disease (Barnett et al., 2012). System‐level therapeutics that target shared biological drivers of ageing could provide a potential solution to this growing burden

The impact of longevity‐enhancing therapies could unfold in several ways. Three broad scenarios illustrate the range of possibilities. All scenarios have been observed in experimental animal studies, although it is often assumed that large leaps in healthspan and lifespan in humans are a distant hope.

Symptom‐management approaches are most likely to produce the outcome in which lifespan increases more than healthspan because they prolong survival without preventing the accumulation of frailty, multimorbidity and disability. Indeed, many developed societies already exhibit this pattern, with survival improving while years lived in poor health continue to expand.

Disease‐specific therapies occupy an intermediate position. They can produce dramatic improvements for individual diseases but are unlikely to generate major increases in population healthspan or lifespan because ageing is characterised by competing and sequential disease risks. For example, even the hypothetical elimination of all cancer has been estimated to increase average life expectancy by only around 3 years, because other age‐related diseases subsequently emerge (i.e., gains are limited by competing risks) (Mackenbach et al., 1999; Naik & Adamic, 2020). Whether such interventions predominantly increase ‘healthy years’ or merely prolong survival depends on two interacting variables: (i) the extent to which the treated disease contributes to morbidity and mortality, and (ii) the nature and timing of subsequent pathologies that emerge thereafter.

System‐level therapies are fundamentally different. Although they remain largely unrealised clinically, recent developments such as GLP‐1 receptor agonists may represent an early manifestation of this emerging therapeutic paradigm. By targeting biological mechanisms shared across multiple age‐related diseases, these therapies are expected to produce pleiotropic benefits across organ systems, analogous to how antibiotics transformed the treatment of numerous infectious diseases by targeting common microbial mechanisms. Consequently, they have the greatest potential to generate rapid, simultaneous improvements in both healthspan and lifespan.

Such interventions are not purely hypothetical. Beyond GLP‐1 receptor agonists, other biological mechanisms have been identified that represent critical system‐level nodes whose modulation could influence multiple age‐related diseases simultaneously. One such example is necrosis, or unregulated cell death, which sits at the intersection of numerous degenerative pathologies. Acute tubular necrosis contributes to kidney disease, neuronal necrosis underlies several neurodegenerative disorders, and related necrotic processes occur across cardiovascular and inflammatory pathologies. Rather than treating these diseases independently, therapeutics targeting necrosis itself could simultaneously alter the trajectories of multiple disorders (Kern, Bonventre et al., 2025). If successful, such therapies would be expected to produce not incremental improvements in individual diseases, but rapid, system‐wide changes in population health.

These distinctions between therapeutic strategies provide the impetus for a simple first‐order conceptual framework linking biological mechanisms to changes in population health. Biological mechanisms are not valued directly; rather, their effects are represented through changes in survival and health‐related quality of life relative to an appropriate counterfactual, for example, existing standard of care. We therefore introduce a formulation for estimating the present monetary value of health‐adjusted survival attributable to a longevity intervention: Incremental present monetary value attributable to a longevity intervention:

(1) $$\Delta PV_{H}=Nv_{H}\int_{0}^{\infty }e^{-rt}\left[S_{1}\left(t\right)q_{1}\left(t\right)-S_{0}\left(t\right)q_{0}\left(t\right)\right]dt$$

ΔPV_H_ is the incremental present value of health; N is the size of the treated population; and t is time from the intervention. The subscripts 1 and 0 denote the intervention and counterfactual scenarios, respectively. $S_{k}\left(t\right)$ is the probability of surviving to time t, while $q_{k}\left(t\right)\in \left[0,1\right]$ represents health‐related quality of life among those alive, where 1 denotes full health and values approaching 0 denote severe impairment. The product $S_{k}\left(t\right)q_{k}\left(t\right)$ represents expected health‐adjusted survival at time t. The parameter $v_{H}$ assigns a monetary value to one health‐adjusted life‐year, expressed in constant‐price currency per health‐adjusted life‐year. Finally, $e^{-rt}$ discounts future health benefits at rate r. The values of $v_{H}$ and r should be selected for the relevant jurisdiction and analytical perspective.

Equation (1) captures improvements in both the duration and quality of life. An intervention creates value when it increases survival, improves health‐related quality of life, or both, relative to the counterfactual. Interventions that delay several diseases would influence both $S_{1}\left(t\right)$ and $q_{1}\left(t\right)$. Population‐level transition speed depends on the timing of uptake. For staggered adoption or heterogeneous populations, Equation (1) can be applied to successive age, risk and treatment cohorts and the resulting values summed. Improvements realised earlier receive greater present weight because they are less heavily discounted. In practical terms, a sudden leap in healthspan and lifespan would not merely extend years lived, but shift large populations into prolonged periods of high‐functioning physiological resilience.

**FIGURE 1:**
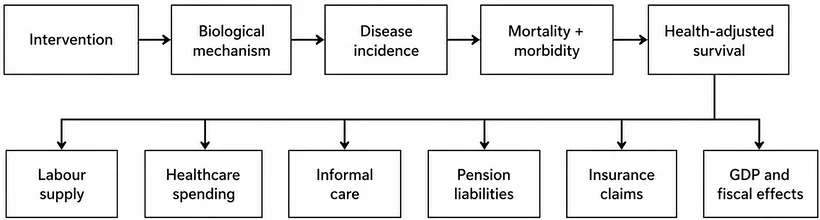
Conceptual framework linking biomedical intervention to health, economic and actuarial outcomes. An intervention acts through biological mechanisms to alter disease incidence and progression, mortality and morbidity, thereby changing health‐adjusted survival. These health effects may subsequently influence labour supply, healthcare spending, unpaid caregiving, pension liabilities, insurance claims, and aggregate GDP and public‐finance outcomes. The direction and magnitude of each downstream effect are context‐dependent and should be estimated separately.

Equation (1) deliberately captures only the monetised health component of an intervention's value. A wider economic assessment would also need to consider treatment and healthcare costs, workforce participation, unpaid care, pension liabilities, insurance exposure and public finances through sector‐specific analyses, as illustrated in Figure 1. Longevity interventions may affect several of these domains simultaneously. Interactions between these effects may be nonlinear and would require additional demographic, fiscal and economic modelling.

## 5 THE LOOMING CHALLENGE FOR FINANCIAL MODELS

For decades, life insurers, pension funds and government actuaries have relied on highly complex, but fundamentally retrospective, models whose foundational assumptions have remained largely unchallenged to predict longevity, healthcare costs and mortality risk. These models assume that ageing follows an irreversible trajectory that steepens with advancing age. As discussed above, increasing evidence from mammalian biology now suggests this assumption may no longer hold. Scientific understanding is advancing rapidly, yet the financial models used to plan for these advances have struggled to keep pace. While important efforts are ongoing to modernise actuarial thinking, there is an urgent need to stop driving the fiscal car by looking only in the rear‐view mirror.

Even the current assumption that past damage from obesity (like prior smoking) may be irreversible, might not hold if new gerotherapeutic drugs prove capable of reversing prior physiological exposures via rejuvenation (i.e., not simply slowing disease progression but reversing it). Consequently, investing in forward‐looking modelling alongside the statistical methods needed to rigorously integrate expert judgement is likely to deliver strong returns, both in accuracy and in policy relevance.

To date, no drugs have demonstrated major extensions in both lifespan and healthspan in humans, so existing models have remained largely unchallenged. Advances such as those emerging from GLP‐1‐based therapies provide a glimpse of what may be possible with system‐level therapeutics, suggesting that transformative breakthroughs in medicine may be closer than many anticipate. When they arrive, the key issue will not be simply ‘how much’ lifespan and healthspan are extended, but ‘how fast’. Are financial systems prepared for the consequences?

## 6 IMPACT ACROSS SECTORS IN RAPID ALTERATIONS OF HEALTHSPAN AND LIFESPAN

The health of the nation has long been a cornerstone of economic growth and productivity. The emerging longevity science revolution will have far‐reaching implications, compelling urgent transformation across multiple sectors:

- Life insurers and pension funds will need to re‐evaluate mortality risk models and capital buffers. Most existing models are designed to accommodate gradual demographic change rather than abrupt biological advances (Table 1). Financial systems might comfortably accommodate a decade increase in life expectancy if it emerges gradually over half a century, but could they accommodate the same increase within 5 years?
- Governments, central banks and financial planners will need to reconsider retirement ages, long‐term care funding, financial stability and the structure of sustainable asset growth in a world where people may live significantly longer. With the right policies in place, the longevity revolution could be a powerful engine for national productivity and economic expansion. Life expectancy nearly doubled in the 20th century, driving unprecedented growth, and history could repeat itself. In other words, the longevity revolution could create a new ‘longevity‐driven economic window of opportunity’, comparable in its long‐term economic significance to periods such as the Industrial Revolution or the post‐Second World War reconstruction boom. The United Kingdom, home to one of the world's most advanced longevity ecosystems, is well‐positioned to lead, but this will require targeted funding and strategic support for life sciences innovation.
- Healthcare systems may ultimately experience substantial reductions in the burden of chronic disease, but the transition itself is unlikely to be straightforward. Health services will need to adapt rapidly by redesigning models of care, reimbursement systems, workforce planning and preventive medicine to reflect populations that remain healthier for longer before experiencing a later onset of disease.

## 7 CONCLUSION

The world is on the cusp of a biological and economic transformation. Advances in science are shifting treatments from reactive disease management to proactive, systems‐level health optimisation. To fully realise the social and economic returns, government and industry must act now. That means funding longevity research at scale, accelerating clinical translation, and modernising the frameworks through which we measure risk and value human health. This is not a distant future; it is already unfolding. The question is no longer if these changes will happen, but how we can harness them to solve one of our most urgent challenges: unlocking productivity in an ageing society.

The emergence of system‐level therapeutics targeting shared upstream drivers of multiple age‐related diseases has the potential to produce rapid, non‐linear improvements in both healthspan and lifespan. Unlike conventional therapies, these interventions could produce rapid shifts in population health and survival, outpacing the assumptions embedded in existing demographic and economic models. Life insurers and pension funds could therefore face rapidly changing mortality assumptions, while governments would need to reconsider retirement ages, fiscal planning, labour markets and healthcare provision, all of which could have destabilising effects on the financial system and economy more broadly. Yet, if anticipated and incorporated into economic planning, these same advances could unlock a substantial window of economic opportunity and offer a direct route to addressing the productivity puzzle in ageing societies.

## AUTHOR CONTRIBUTIONS

All authors contributed equally to the manuscript. Carina Kern conceptualised and wrote the first draft, Nikodem Grzesiak and Damian Miles Bailey formalised the conceptual and quantitative framework, and Serena Kern‐Libera, Guy Coughlan, Richard Faragher and Annalisa Jenkins contributed equally to the development and revision of the manuscript. All authors have read and approved the final version of this manuscript and agree to be accountable for all aspects of the work. All persons designated as authors qualify for authorship, and all those who qualify for authorship are listed.

## CONFLICT OF INTEREST

C.K., S.K‐L. and N.G. are CEO, COO and CTO of LinkGevity, respectively. D.M.B. is Editor‐in‐Chief of *Experimental Physiology*, Chair of the Life Sciences Working Group, member of the Human Spaceflight and Exploration Science Advisory Committee to the European Space Agency, member of the Space Exploration Advisory Committee to the United Kingdom and Swedish National Space Agencies and member of the National Cardiovascular Network for Wales and South‐East Wales Vascular Network. D.M.B. is also affiliated to Bexorg, Inc. (USA) focused on the technological development of novel biomarkers of cerebral bioenergetic function and structural damage in humans.

## FUNDING INFORMATION

This work did not receive any funding from public, commercial, or not‐for‐profit organizations. D.M.B. is supported by a Royal Society Wolfson Research Fellowship (Grant No. WM170007).

## GENERATIVE AI STATEMENT

Generative AI assistance (OpenAI ChatGPT) was used to check and improve the grammar, language, readability, and clarity of the manuscript. All scientific content, interpretations, and conclusions were developed and verified by the authors, who take full responsibility for the content of the publication.
